# Puerperal Insanity

**Published:** 1877-09

**Authors:** V. H. Taliaferro

**Affiliations:** Atlanta, Ga., Professor of Obstetrics and Diseases of Women and Children, Atlanta Medical College; President of the Atlanta Academy of Medicine


ATLANTA

MEDICAL AND SURGICAL JOURNAL

VOL. XV.] SEPTEMBER—1877. [No. 6

### Original Communications.

### PUERPERAL INSANITY.

BY V. H. TALIAFERRO, M.D., ATLANTA, GA.,
Professor of Obstetrics and Diseases of Women and Children, Atlanta Medical College;
President of the Atlanta Academy of Medicine.

Mrs. ——, a young, healthy primipara, was delivered at term of a vigorous child, after an unusually severe and exhaustive labor. Save in its protracted severity, the labor was in every respect natural. The external soft parts were unusually rigid and unyielding, holding the head for several hours upon the floor of the pelvis, against the most rapid and powerful throws of labor. It was feared, and indeed expected, that the perineum would give way by rupture. This, however, did not occur, and the labor was terminated without accident to mother or child. For some days following, nothing unusual was observed unless it was in the *remarkably* good condition of the mother, after so severe and protracted labor. She expressed herself in the most cheerful and gratified manner, as perfectly well, and free from any feeling of oppression or exhaustion. The lochia was normal, the appetite good, and the sleeping good until the third night, when she complained that she had slept badly, though entirely free from the least restlessness, pain or uneasiness. On the following (4th) night, being again wakeful, she took, as directed, two small doses of bromide of potassium and chloral, after which she slept well, and awoke refreshed in the morning.

On the third and fourth days following the labor it was observed that there was not the usual accession of milk and distention of the breasts, and the mother complained at not having nourishment for her babe. No especial importance, however, was attributed to this, as it was ascertained that the nurse had applied the child to the breasts at *very long intervals*, upon the supposition that "there was nothing for it to get." The naturally large size of the mammary glands and the general physique of the patient gave every reason to suspect an early accession of milk, upon the natural stimulation of the glands by the frequent application of the child.

Up to this time, the fourth day after labor, two incidents only are worthy of note: the entire freedom from discomfort, with the rather extravagant expressions of the patient as to her *excellent condition*, and the absence of the usual active secretion of milk. About this time some little mental worry and anxiety was manifested at the absence of the milk, and the probable necessity of having to resort to artificial feeding of the infant.

The patient still continued to sleep badly, and taking a dislike to the bromide and chloral, morphine in fourth-grain doses was substituted with satisfactory results. So the case progressed until the sixth day following the labor, when the nurse, in whom she had great faith and confidence, left her to take charge of another case. This caused her much displeasure and worry, and she talked about it a great deal. Another nurse was obtained, to whom she soon took a great dislike, and in a few days discharged her. She now talked and worried almost constantly about her great trouble with her nurses, and it became manifest that there was a morbid mental condition, and the husband was notified of the probable danger of *puerperal insanity.*

From day to day the mental disturbance increased until *insanity* was unmistakeably established. The patient now talked incessantly and with great rapidity, except when asleep with opiates. The insomnia became obstinate and persistent, and the doses of morphine increased to one-half grain, and two or three doses given before getting the desired effects. Up to this time the physiological condition, aside from the mental manifestations, was good save in a *too rapid pulse.* In some ten or twelve days of the illness the pulse ran up to 135 or 140 to the minute, very feeble, and with considerable nervous prostration. Dr. W. F. Westmoreland was now called in consultation, and concurred as to the diagnosis and plan of treatment instituted. The abdomen was found slightly distended and tympanitic, and she was ordered a dose of saccharated calomel, which acted well, relieving the distention and tympanitis. Opium and chloral, which she had been taking, were continued, with the result, generally, of a good night's sleep, though the quantity necessary to attain this end, was increased every day or two. The rapid pulse and nervous prostration were greatly relieved, but without a corresponding mental improvement. From this time the treatment consisted of nourishment freely administered—the bowels occasionally emptied by saccharated calomel—morphine hypodermically, and chloral by the mouth, for some two weeks, when, the symptoms again becoming more grave, Dr. Simpson was called in consultation. The morphine and chloral no longer controlled, satisfactorily, the insomnia, and it was decided to give her bromide of lithia, which was administered in thirty-grain doses. This, after a few doses, was abandoned, because of its rejection by the stomach, and bromide of potassium substituted, in sixty-grain doses, by the rectum, every two hours, until three hundred grains were administered. The whole quantity was perfectly retained, and the patient slept for some ten or twelve hours.

The rectum now ceased to retain the bromide, and it was reluctantly discontinued, the stomach, also, being too irritable to tolerate the large doses necessary. The bromide was resorted to by the rectum at the time, because of the increased obstinacy of the insomnia, and the evident unpleasant effects of the large doses of opium and chloral which had become necessary to induce sleep. The powers of the bromide, as a hypnotic, were clearly demonstrated in this case—the patient going twenty-four hours without any other sedative, and having the best night's sleep after its use she had had since her sickness. Following its use, she ceased her incessant talking, her mental condition appeared improved, and strong hopes were entertained of a favorable turn in her condition. Such hopes, however, were of short duration. Frequently, during her sickness, the mind would clear up for hours, and sometimes for the entire day, when it would seem from her condition, both mentally and physically, that the dark cloud had really passed away, and no obstacle barred a rapid restoration to health. But alas! without warning, and in an instant of time, all would be changed, the most violent insanity re-asserting its complete supremacy.

A little more than a month from the time of the patient's confinement her condition changed decidedly for the worse. The pulse increased in frequency, the respiration, heretofore good, became excited, and the temperature, which had hardly gone over 102 was now 103¼ Far. Dr. W. F. Westmoreland, who had been absent from the city, and now returned, saw her again, and, although her condition was grave in the extreme, he felt hopeful as to the final result, believing the grave symptoms most likely dependent upon meningial inflammation of the brain, which it was hoped would be relieved by a large cantharidal blister upon the back of the neck, extending down the spine some six or eight inches. The blister was applied with some temporary improvement, but she soon was worse, and died on the second day after the application of the blister.

The literature of puerperal insanity affords us but little satisfaction as to the nature of the affection. Burns, who wrote in 1813, recognized different forms of puerperal mania. He speaks of the disease being "*dependent on a morbid irritation, such as inflammation of the mamma, etc.,*" and says that, "here our attention must be directed to the causes." Again, he says: "But I believe it never becomes permanent, nor does it prove fatal unless dependent on phrenitis. * * * * It (*phrenitis*) must be distinguished from from puerperal delirium, which is seldom dangerous, whilst this (*phrenitis*) is a most fatal disease.

Davis, cotemporary with Burns, assumes, likewise, the inflammatory nature of puerperal mania.

Rob't Gooch, the distinguished obstetrician, author and teacher, whose lectures in St. Bartholomew's Hospital, were published after his death, (the third American edition in 1840) says: "When puerperal mania does take place, the patient swears, bellows, recites poetry, talks bawdy, and kicks up such a row that there is the devil to pay in the house; it is odd that women who have been delicately brought up, and chastely educated, should have such rubbish in their minds." * * * * "The fatal cases seem to be those in which there is considerable disturbance of the circulation of the membranes of the brain; I have seen three cases of this kind; but those dependent merely upon nervous irritation do well." Thus it appears that Gooch also recognises several varieties of this disease.

Tyler Smith writing in 1858, says: "Genuine mania, or that which occurs shortly after parturition, appears to me to depend, in the great majority of cases, upon the shock of labor; the general and local irritation incident to delivery, and its immediate consequences; and upon the exhaustion produced by the accumulated effects of gestation and parturition. Except in the few cases in which inflammation of the brain occurs, when it is removed from ordinary insanity, the disease is, I believe, always attended by constitutional exhaustion."

Churchill in 1857, says: "The exciting causes are said to be fright, anger, sorrow, or any species of mental emotion," etc. * * * * "There is no reason to believe that it arises from inflammatory action in the brain." * * * * * "It mayterminate in death. This is almost peculiar to those cases where the pulse is quick, and fever is present."

Ramsbotham in 1860, writes of puerperal mania as follows: "To distinguish it from phrenitis is more difficult; as inflammation of the brain, however, generally appears sooner after labor than true mania, the period of the attack will in some degree guide us. The delerium attendent on phrenitis is preceded by fever and pain in the head, of a longer or shorter duration, with vertigo, singing in the ears, and a flushed cheek; incoherence in manner and expression is often the first symptom observed in mania. Sight and sound can scarcely be borne in phrenitis; they make little unusual impression on the maniacal patient. Inflammatory fever and increased heat of surface, accompany phrenitis in a high. degree; in mania the former of these is absent altogether; and in the latter, if present, is but slight in intensity."

Leishman in 1873, says: "Although, therefore, we admit phrenitis' to be classed as a possible complication of the puerperal state, there is little likelihood of our diagnosis being obscured by such an occurrence. The very early period of its accession after delivery, and the manifestation of headache, suffusion of the eyes, and other local symptoms, referable to the head, would doubtless indicate the nature of the disease to the judicious practitioner. But not only do we discard the idea of imflammation as pathognomonic of puerperal mania, but we embrace without hesitation a directly opposite view, that it is essentially a disease of exhaustion."

Sir J. Y. Simpson in his clinical lectures on diseases of women, speaking of "*a complication of inflammatory diseases in the puerperal state,* says: "To this variety of mental disorder, which is no real form of puerperal mania, I refer now merely to warn you against the error of supposing that puerperal mania itself depends on any inflammatory affection of the brain or its membranes. Meningitis or phrenitis may come to be set up in any case of puerperal mania, and to increase in no small degree the patient's danger; but as was first clearly pointed out by Dr. Gooch, there is no necessary connection between the two forms of disease, and even their association in any case is but a rare coincidence." Prof. Simpson, as early as 1856, brought to the attention of the Edinburgh Obstetric Society his observations on the connection of albuminuria with puerperal insanity in four successive cases. In his work upon diseases of women, he says: "*a.* That albuminuria precedes and attends the first access of puerperal insanity in a large proportion of cases; but perhaps not so frequently and so constantly as it precedes and attends upon attacks of puerperal convulsions. I have found it present in eight out of ten cases of puerperal insanity at the commencement of the disease; and possibly it escaped observation in these two cases from not being looked for sufficiently in their progress. For it seems to me: *b.* That the coagulability of the urine in puerperal insanity generally disappears within a short time after the attack commences, and hence disappears more speedily than happens in puerperal convulsions. The fire of this disease goes on burning in these cases of insanity after the lighted match is merely applied, and the strange morbid clockwork runs on, as it were, after the key that wound it up is withdrawn. I have seen all traces of albuminuria in puerperal insanity disappear from the urine within fifty hours, from the accession of the malady. The general rapidity of its disappearance is, perhaps, the principle or, indeed, the only reason why this complication has escaped the notice of those physicians among us who devote themselves with such ardor and zeal to the treatment of insanity in our public asylums."

Prof. Simpson again says: "In the blood of the puerperal female—greatly modified as it is in the normal states of pregnacy and delivery, and containing as it does after parturition the effete elements of the involving or disentegrating uterus, and the materials for the new lochial secretion—ferments, and agents may possibly exist, which are more apt to develop special morbid poisons out of the retained renal excretions than happens in other states of the system. But I repeat, the whole subject is yet quite dark and conjectural, and will remain so till pathological chemistry is able to cast some light upon it."

Fordyce Barker, in his work on puerperal diseases in 1874, gives as the most prominent *predisposing* causes: 1st. Hereditary tendency; 2d. Dystocia. In reference to the exciting causes he says: "It is my firm conviction that mental emotions constitute the exciting cause of puerperal mania infinitely more frequently than all other causes combined." In proof of this assertion the relative frequency of puerperal mania in a number of lying-in hospitals is given.

In Wiirzburg, the proportion of cases of mania to the whole number of confinements was 1 in 1,487; in Prague, 1 in 1,228; in St. Giles Infirmary, 1 in 1,888; in Queen Charlotte's Lying-in Hospital, 1 in 182.

In explanation of the marked increase in the percentage of cases in the latter institution, Prof. Barker says: "A large majority of patients in our lying-in wards are of foreign birth. They have come to a new country, leaving friends behind, with the hope of improving their condition, and many are disappointed in this respect. A large proportion probably one-half are unmarried." "Very many of these are suffering the disgrace of seduction and abandonment. In addition to this, I am well convinced that our climate has a marked influence in developing the nervous susceptibility of Europeans who come here. Then, again, there is no part of the world where the lapse from virtue in women is so surely punished by social ostracism as in New England, and she contributes her quota of poor girls who rush to a great city to hide themselves, and are at last driven to the hospital as their only recourse."

Prof. Barker thinks if statistics prove anything, "they prove that moral emotions are the great exciting cause of puerperal mania."

The social and domestic relations of the case I have detailed here were all that could be desired, and therefore preclude the probability of these having any agency in her insanity. Yet the starting point of the mental disturbance was manifested upon the loss of her nurse, to whom she was greatly attached in the first place, and subsequently agravated by a nurse to whom she took a great dislike, and who told her alarming stories of the sufferings and dangers of lying-in women.

Barker and Leishman, who have given this subject of late years careful and earnest attention, differ but little as to the causation—the one looking to the mental and moral emotions, and the other chiefly to nervous exhaustion; indeed Churchill, Tyler Smith, and Ramsbotham are much on the same line.

With all the lights before us, it would seem that a combination of circumstances most likely exist to give rise to puerperal mania: 1st. A predisposition, hereditary or acquired; 2d. Nervous exhaustion; 3d. Mental or Moral emotions; and possibly, 4th. Poisonous or excrementitious material in the blood from the involving uterus, or from the kidneys. Two or more of these may be sufficient to give rise to the disorder, and again the force of the entire combination may be necessary.

It is well known that hysteria is the product, most usually, of uterine irritation, but it by no means follows that every woman with uterine irritation must have hysteria. Why, from the same cause, some have hysteria and others do not, we can explain in no other way than by a peculiar nervous organization or susceptibility—these being congenital, or acquired by habits of life, etc. So with puerperal mania, while many resist the combination of causes mentioned, the few, from some unrecognized and unknown predisposition or nervous susceptibility, yield.

The insanity of pregnancy, and the insanity of lactation, must not be confounded with *puerperal mania.* This disorder, as indeed all forms of insanity may, and not unfrequently do, comepletely mask grave organic diseases. Upon this point Prof. Barker gives us the following precaution: "In this hospital (Bellevue) one patient has died with pelvic peritonitis, another with pneumonia, and a third with pericarditis and endocarditis, and in neither was the disease suspected until revealed by autopsy." In the prognosis, we are told by Gooch, William Hunter, Simpson, and others, that excessive vascular action portends danger to life. We know that excessive vascular action portends organic lesion; therefore, a rapid pulse would seem to indicate organic complications as the real seat of danger to life. We must conclude, then, that insanity, whether puerperal or otherwise, cannot become grave in reference to life without organic lesion. It is true, we may have death from nervous shock, nervous exhaustion, etc., but certainly not from the shock or exhaustion that fails to be manifest for days. In the case presented, the physical condition was fine both before and after labor. There was no loss of blood. There was no accident or circumstance calculated to exhaust the powers of life, save in the *hard labor.* The patient was in splendid health, and reacted at once from the shock and exhaustion of labor. In a healthy subject we are not to expect serious exhaustion from the pains of labor, though severe and protracted. The pain is physiological, and, ordinarily, not exhaustive. Hence, we are unable to accept, in toto, the *exhaustive theory* of the distinguished authors quoted.

We are driven to the conclusion that, in the case reported, there was organic disease masked by the insanity so completely that nothing short of an autopsy could possibly have revealed the true pathological condition and cause of death.

